# Avermectins Inhibit Replication of Parvovirus B19 by Disrupting the Interaction Between Importin α and Non-Structural Protein 1

**DOI:** 10.3390/v17020220

**Published:** 2025-02-03

**Authors:** Gualtiero Alvisi, Elisabetta Manaresi, Silvia Pavan, David A. Jans, Kylie M. Wagstaff, Giorgio Gallinella

**Affiliations:** 1 Department of Molecular Medicine, University of Padova, 35121 Padova, Italy; silvia.pavan.1@unipd.it; 2 Department of Pharmacy and Biotechnology, University of Bologna, 40138 Bologna, Italy; elisabetta.manaresi@unibo.it; 3 Department of Biochemistry and Molecular Biology, Biomedicine Discovery Institute Monash University, Clayton, VIC 3800, Australia; david.jans@monash.edu (D.A.J.); kylie.wagstaff@monash.edu (K.M.W.)

**Keywords:** ivermectin, avermectins, AlphaScreen, antivirals, parvovirus B19, importin alpha, NS1, nuclear transport

## Abstract

Human parvovirus B19 (B19V) is a major human pathogen in which the ssDNA genome is replicated within the nucleus of infected human erythroid progenitor cells (EPCs) through a process involving both cellular and viral proteins, including the non-structural protein (NS)1. We previously characterized the interaction between NS1 classical nuclear localization signal (cNLS: GACHAKKPRIT-182) and host cell importin (IMP)α and proposed it as a potential target for antiviral drug development. Here, we further extend on such findings. First, we demonstrate that NS1 nuclear localization is required for viral production since introducing the K177T substitution in a cloned, infectious viral genome resulted in a non-viable virus. Secondly, we demonstrate that the antiparasitic drug ivermectin (IVM), known to inhibit the IMPα/β dependent nuclear import pathway, could impair the NS1-NLS:IMPα interaction and suppress viral replication in UT7/EpoS1 cells in a dose-dependent manner. We also show that a panel of structurally related avermectins (AVMs) can dissociate the NS1-NLS:IMPα complex with half-maximal inhibitory concentrations in the nanomolar range. Among them, Eprinomectin emerged as the most selective inhibitor of B19V replication, with a selectivity index of c. 5.0. However, when tested in EPCs generated from peripheral blood mononuclear cells, which constitute a cellular population close to the natural target cells in bone marrow, the inhibitory effect of IVM and Eprinomectin was demonstrated to a lesser extent, and both compounds exhibited high toxicity, thus highlighting the need for more specific inhibitors of the NS1-NLS:IMPα interaction.

## 1. Introduction

Human parvovirus B19 (B19V) is a widely diffused pathogen that is classified in the *Erythroparvovirus* genus within the *Parvoviridae* family [1,2]. It commonly infects people at a young age, often leading to fifth disease, also known as *erythema infectiosum*, a mild childhood illness, or causing joint issues in adults [3,4]. Furthermore, it can cause serious problems, including both acute and chronic red blood cell deficiency in individuals with erythropoietic disorders and/or immune system deficiencies due to its ability to infect and induce apoptosis in erythroid progenitor cells (EPCs) [5]. Finally, infection during pregnancy can lead to severe outcomes like fetal hydrops and even death [6]. B19V is a small, non-enveloped virus with a 5.6 Kb single-stranded DNA genome. The genome has palindromic sequences at both ends, which are essential for replication and form hairpin structures serving as the origin of replication. Among the proteins encoded by the virus, the best-known ones are the capsid proteins VP1 and VP2 [7] and the nonstructural protein (NS)1 [8]. B19V is believed to successfully replicate exclusively in dividing EPCs found in the bone marrow and fetal liver [9], where it halts the cell cycle and induces apoptosis [10]. This limited tropism represents a major hurdle for studying the virus in cell culture, with human primary CD36+ EPCs and the UT7/EpoS1 cell line being the most widely used models [11,12,13]. Replication of the viral genome occurs in the nucleus of host cells, likely through a rolling hairpin mechanism, which involves DNA repair processes facilitated by both the host cell machinery and the viral protein NS1 [14]. NS1, a 74 kDa protein composed of 671 amino acids, has critical roles in viral replication, possessing helicase, endonuclease [15], and transactivation activities [16], as well as profoundly modulating cell functions [10,12,17,18,19,20]. Since no vaccines or specific antiviral treatments have been approved to target B19V replication so far, identifying new therapeutic targets is of high importance [21], and NS1 can be such a relevant target. Given its size, NS1 must be actively transported into the nucleus to function. We have recently shown that NS1 is imported in the nucleus by the importin (IMP)α/β heterodimer, whereby IMPα recognizes a classical nuclear localization signal (NLS) located at residues 172–182 (172-GACHAKKPRIT-182). The K177T substitution ablated interaction of a B19V NLS peptide with recombinant IMPα in vitro and nuclear localization and viral transcription in a minigenome system [22]. Given the evidence that Ivermectin (IVM), an FDA-approved anthelmintic drug [23], which has been shown to interfere with IMPα/β heterodimer stability and function [24], also inhibited viral replication in cell culture [22], we decided to further validate NS1 nuclear import as an antiviral target. We show here that NS1 nuclear import is required for viral replication in cell culture in the context of a full-length viral genome. Secondly, we showed that IVM significantly reduced the NS1:IMPα interaction. IVM could inhibit B19V replication in UT7/EpoS1 with a selectivity index (SI) of 1.9, indicating a small therapeutic window. However, several avermectins (AVMs), closely related to IVM, hindered the NS1:IMPα interaction similarly to IVM. Among them, Eprinomectin exhibited a SI higher than IVM (about 5.0). However, when tested in the more physiological EPCs, both IVM and Eprinomectin proved highly toxic. Therefore, although the inhibition of NS1 nuclear import appears to be a viable target for antiviral therapy, more selective inhibitors of the NS1:IMPα interaction are needed to treat B19V-infected patients.

## 2. Materials and Methods

### 2.1. Plasmids

Plasmid pCK10, containing a genomic insert extending from position 136 to position 5461 of the B19V EC consensus sequence, was previously described [25]. This plasmid includes the complete internal region and extension of both inverted terminal repeats (ITRs) beyond the sites of dyad symmetry, with maintenance of the potential to fold back in hairpin structures and preservation of flip/flop sequence heterogeneity. Plasmids pCK10-NS1-ATG-KO, whereby the first start codon of full-length NS1 was mutagenized to prevent expression of full-length NS1, and pCK10-NS1;K177T bearing a Thr in place of the key NLS residue Lys177 were generated by site-specific mutagenesis using plasmid pCK10 as a template (BioFab research, Rome, Italy). Plasmid pGEX-2T/mIMPα2(70-529), encoding for mouse IMPα2 devoid of the autoinhibitory IMPβ binding domain (mIMPα2ΔIBB), was previously described [26].

### 2.2. Cells

UT7/EpoS1 cells were cultured in IMDM (Cambrex, Paullo, Milan, Italy), supplemented with 10% fetal calf serum (FCS) and 2 U/mL Epoetin beta (NeoRecormon, Roche, Monza, Italy), at 37 °C and 5% CO_2_. Cells were kept in culture at densities between 2 × 10^5^–1 × 10^6^ cells/mL and used for transfection experiments when at a density of 3 × 10^5^ cells/mL. EPCs were generated in vitro from peripheral blood mononuclear cells (PBMCs) obtained from the leukocyte-enriched buffy coats of healthy blood donors, available for institutional research purposes according to the policy approved by the local Ethical Committee (S.Orsola-Malpighi University Hospital). PBMCs were isolated using centrifugation in Ficoll-Paque Plus (GE Healthcare Bio-Sciences AB, Milan, Italy) and cultured in a medium containing erythropoietic growth and differentiation factors, following previously established protocols with minor modifications [11]. Isolated PBMCs were cultured in IMDM supplemented with 20% serum substitute BIT 9500 (StemCell Technologies, Vancouver, BC, Canada) and enriched with 900 ng/mL ferrous sulfate, 90 ng/mL ferric nitrate, 1 µM hydrocortisone (#H0888, Merck Millipore, Milan, Italy), 3 U/mL Epoetin beta (NeoRecormon, Roche, Monza, Italy), 5 ng/mL IL-3 and 20 ng/mL stem cell factor (Thermo Fisher Scientific, Monza, Italy). Cells were used for infection experiments after 8 days of in vitro growth and differentiation.

### 2.3. Transfection for Generation of Infectious Viruses

Linearized B19V viral genomes were obtained by the digestion of pCK10 plasmids with SacI (#FD1133; Thermo Fisher Scientific, Monza, Italy). UT7/EpoS1 cells were transfected by using the Amaxa Nucleofection System (Lonza, Siena, Italy), with V Nucleofector Reagent and T20 program setting, at a ratio of 1 µg insert DNA for 10^6^ cells. Following transfection, the cells were incubated at 37 °C in complete medium at an initial density of 10^6^ cells/mL. Constant amounts of cell cultures were collected up to 6 days post-transfection (dpt); cells and cell-free supernatants were separated by centrifugation at 2350× *g* for 5 min then used for analysis and/or successive infection experiments [25].

### 2.4. Infection of EPCs with Cell Supernatants

Cell-free supernatants obtained from nucleofected UT7/EpoS1 cells were used to infect 10^6^ EPCs at a ratio of 2.45–2.82 Log genome equivalents (geq)/cell [25]. The infection was carried out at 37 °C for 2 h. Then, cells were washed free of inoculum and expanded in complete medium at an initial density of 10^6^ cells/mL. Constant amounts of cell cultures were collected at 2 and 48 h post-infection (hpi), and at 6 days post-infection (dpi), cells and cell-free supernatants were separated by centrifugation at 2350× *g* for 5 min then used for analysis.

### 2.5. Nucleic Acids Purification

Equal amounts of cell cultures, corresponding to 1 × 10^5^ cells, collected at the appropriate time points following infection were processed by using the Maxwell Viral Total Nucleic Acid kit (#AS1330, Promega Italia, Milan, Italy) on a Maxwell MDx platform (Promega, Italia, Milan, Italy), following the manufacturer’s instructions, in order to obtain a total nucleic acid fraction in elution volumes of 100 µL. Volumes of 10 µL were then used in the subsequent qPCR assays for the quantitative evaluation of target DNA.

### 2.6. Quantitative PCR

Viral DNA was amplified by using the primer pair R2210-R2355, located in the central exon of the B19V genome [27]. As a control, a target sequence in the region of genomic DNA coding for the 5.8 S rDNA gene was amplified by using the primer pair 5.8S_S1 (CTCTTAGCGGTGGATCACTC)—5.8S_A1(GTGCGTTCGAAGTGTCGATG). All oligonucleotides were obtained from Eurofins Genomics (Milan, Italy). Quantitative PCR was carried out using the RotorGeneQ System (Qiagen, Milan, Italy) and SybrGreen detection of amplification products. Amplification reactions were performed using Maxima SYBR Green qPCR Master Mix (Thermo Fisher Scientific, Monza, Italy) including 16 pmol of each specific primer pair. For PCR, the thermal profile consisted of 10 min at 95 °C then 40 cycles of 15 s at 95 °C, 30 s at 60 °C, and 30 s at 72 °C coupled with signal acquisition. A final melting curve was performed, with the thermal profile ramping from 65 °C to 95 °C at a 12 °C/min rate, coupled with continuous signal acquisition. Fluorescence emission recorded in the FAM/Sybr channel of the instrument was analyzed by using the functions available in the RotorGene 6.0 software (Qiagen, Milan, Italy). All reported experiments were carried out in triplicate, each sample analyzed in duplicate, and melting curve and quantitative analysis performed. Melting curve analysis was used to determine the specificity of the amplification products by defining, for each reaction, the melting profile, and the Tm of the products. Absolute quantitation of viral DNA was obtained by calibration to standard targets prepared from cloned templates. Data analysis was carried out by using the program GraphPad Prism 10.0 (Dotmatics, Boston, MA, USA).

### 2.7. Indirect Immunofluorescence (IIF) and Confocal Laser Scanning Microscopy (CLSM)

To detect viral proteins by IIF, 5 × 10^4^ transfected UT7/EpoS1 were spotted on glass slides and fixed with 1:1 acetone/methanol for 10 min at −20 °C. For detection of NS1, cells were incubated with undiluted supernatants from hybridoma #892/5, containing a monoclonal mouse antibody directed against NS1 [28]. To detect structural proteins VP1/2, cells were incubated with an anti-VP1/2 mouse monoclonal antibody (#8293, Chemicon, Merck Millipore, Milan, Italy; 1:200 in PBS/BSA 1%). Following three washes in PBS, cells were further incubated with an anti-mouse Alexa Fluor 488-conjugated secondary antibody (#A-11001, Thermo Fisher Scientific, Monza, Italy; 1:1000 in PBS/BSA 1%). Nuclei were stained with Hoechst 33′342 (#62248, Thermo Fisher Scientific, Monza, Italy; 20 μM in PBS). Five random fields per condition were acquired using a Nikon AX-R confocal laser scanning microscope (Nikon Europe B.V., Amstelveen, The Netherlands) equipped with a 20× objective and appropriate laser lines [29]. The number of positive cells was analyzed using the program QuPath v0.5.1 [30].

### 2.8. Drugs

Importazole (#S8446), Avermectin B1 (#S4999), Eprinomectin (#S3591), Selamectin (#S5247), Emamectin benzoate (#S4423), Milbemycin oxime (#S5055), Moxidectin (#S3713), and Ivermectin (#S1351) were purchased from Selleckchem (Houston, TX, USA). All compounds were resuspended in DMSO 100% (*v*/*v*) at a final concentration of 50mM and kept at −20 °C until needed.

### 2.9. Cell Cytotoxicity Assays

The assessment of cellular viability and metabolic function in UT7/EpoS1 cells and EPCs was carried out through the measurement of intracellular reducing activity using the alamarBlue™ HS Cell Viability Reagent (#A50100, Thermo Fisher Scientific, Monza, Italy) or via a WST-8 based assay (Cell Counting Kit-8, CCK-8, Dojindo Molecular Technologies, Rockville, MD, USA), respectively. Cells were seeded at the density of 5 × 10^4^ cells/100 µL in a 96-well plate, cultured for 6 h in complete growth medium supplemented with different concentrations of compounds, and then washed in PBS and cultured for a further 48 h in the presence of compounds. After 44 h, the alamarBlue™ HS Cell Viability Reagent or the CCK-8 solution was added, and cellular viability and metabolic function were measured according to the manufacturer’s instructions. Data were expressed as mean percentage values of treated cells relative to untreated controls. Half maximal cytotoxic concentrations (CC_50_) were calculated using the software GraphPad Prism version 10.0 (Dotmatics, Boston, MA, USA) to analyze the nonlinear regression and plotting log inhibitor concentration against the normalized response.

### 2.10. Antiviral Assays

B19V was obtained from a cloned synthetic genome, as described [25]. For infection, UT7/EpoS1 cells or EPCs were incubated in PBS at a density of 1 × 10^7^ cells/mL in the presence of B19V at a multiplicity of infection of 10^3^ geq/cell. Following 2 h at 37 °C, cells were washed twice in PBS to remove the inoculum virus and incubated at 37 °C in 5% CO_2_ in the complete growth medium at an initial density of 1 × 10^6^ cells/mL. For the investigation of antiviral properties activity, cells were cultured for 6 h in a complete growth medium supplemented with different concentrations of compounds, washed in PBS, infected as described, and cultured for a further 48 h in the presence of compounds. The extent of viral replication was measured as the increase in Log B19V DNA from 2 to 48 hpi in untreated and treated cells. The antiviral activity of a compound was expressed as the mean ratio value of Log increase in treated cells relative to untreated control. Half maximal effective concentrations (EC_50_) were calculated using the software GraphPad Prism version 10.0 (Dotmatics, Boston, MA, USA) to analyze the nonlinear regression and plotting log inhibitor concentration against the normalized response. The SI relative of each compound was calculated as the ratio between CC_50_ and EC_50_.

### 2.11. Protein Purification and Biotinylated Peptides

GST-mIMPα2ΔIBB was purified from bacteria as a GST-fusion protein under native conditions as described in [31]. Peptides biotinylated at the N-terminus were purchased from Genscript (Singapore). Sequences utilized were Simian Virus (SV)40 Large Tumor Antigen (LTA) NLS wt: SQHSTPPKKKRKV; SV40 LTA NLS mut: SQHSTPPKtKRKV; B19V NS1 NLS wt: GACHAKKPRIT; B19V NS1 NLS mut: GACHAtKPRIT.

### 2.12. AlphaScreen-Based Binding Assay

The AlphaScreen assay was performed in quadruplicate in 384-well white opaque plates (Revvity, Waltham, MA, USA) as previously described [31]. Briefly, 120 nM final concentration of the B19V NS1 biotinylated wt or mut NLS peptides or 30 nM final concentration of the SV40 LTA NLS wt or mut biotinylated NLS peptides was added to each well, followed by appropriate concentrations of the GST-tagged mIMPα2ΔIBB (ranging from 0 to 60 nM, 12 concentrations total), prepared by serial dilution in PBS, and incubation for 30 min at room temperature. All subsequent additions and incubations were made under subdued lighting because of the photosensitivity of the beads. 5 μL of a 1:50 dilution (in PBS containing 0.5% BSA) of anti-GST acceptor beads was added and incubated for 90 min at room temperature. Then, 5 μL of a 1:50 dilution of the streptavidin donor beads was added to provide a final sample volume of 25 μL, and the mixture was incubated at room temperature for 2 h. The assay was quantified on a Pherastar plate reader (BMG LABTECH, Ortenberg, Germany) with an AlphaScreen detection kit, quadruplicate values were averaged, and titration curves (sigmoidal dose response) were plotted using GraphPad Prism version 10.0 (Dotmatics, Boston, MA, USA). Values in the ‘hooking zone’, where quenching of the signal has occurred through the presence of too much of either binding partner, were excluded from the final plot as previously [31]. These graphs were used to determine the screening concentration for the AVM inhibition tests. Inhibition assays were performed using AlphaScreen as above with the following adjustments: 5 μL serial dilutions of each drug compound (made up of 0.05% PBS DMSO) were added to the well first. This was followed by a 60 nM final concentration of biotinylated peptide and a 30 nM final concentration of GST-tagged mIMPα2ΔIBB and incubated 30 min at room temperature. Acceptor/Donor bead additions and plate reading/analysis to determine the half-maximal inhibitory concentration (IC)_50_ were performed as per the standard assay above.

### 2.13. Bioinformatics

The presence of putative alternative translation initiation sites for NS1 was assessed by analyzing the sequence of pCK10 using software ATGpr (https://atgpr.dbcls.jp) [32], using standard parameters and a threshold score of 0.09. Only translation initiation sites using the same frame as full-length NS1 were considered.

## 3. Results

### 3.1. NS1 Nuclear Localization Is Absolutely Required for Viral Particle Production

**Figure 1:**
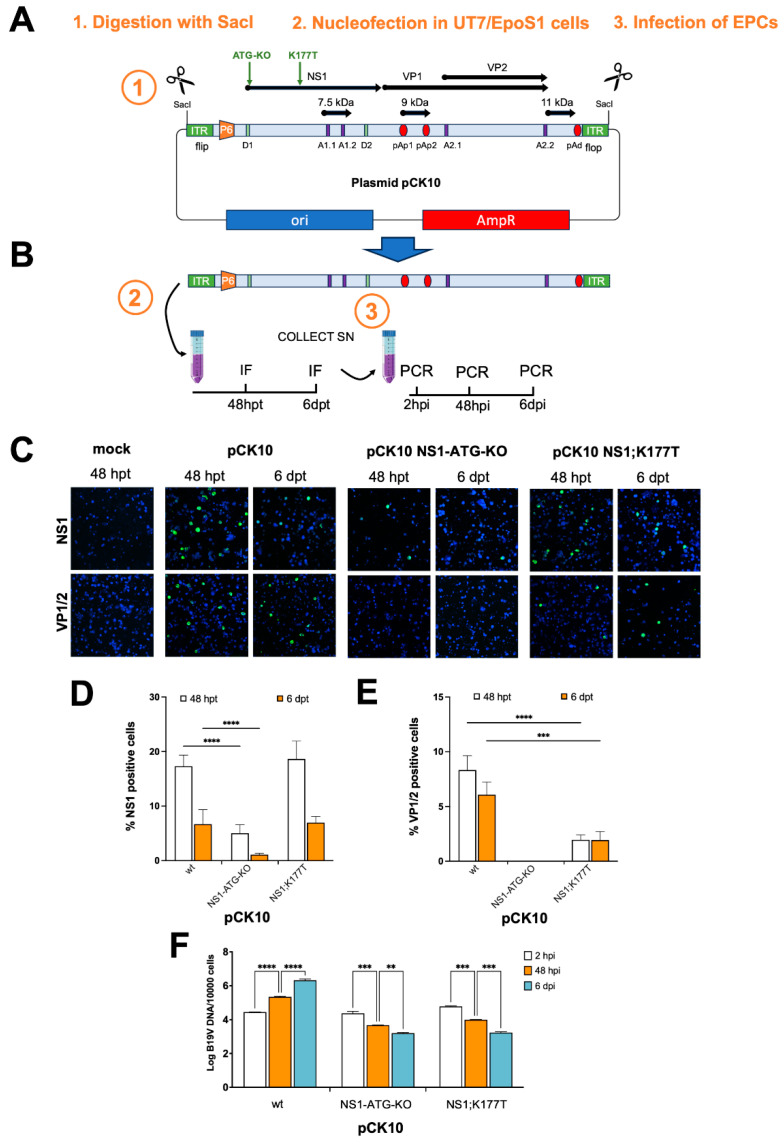
B19V NS1 NLS is essential for viral replication. (**A**) Schematic representation of pCK10 plasmid and derivatives thereof containing a synthetic consensus B19V genome with inverted terminal repeats (ITRs) in a flip/flop configuration flanked by SacI sites and ORFs represented by black horizontal lines. P_6_, promoter; pAp1, pAp2, proximal cleavage-polyadenylation sites; pAd, distal cleavage-polyadenylation site; D1, D2, splice donor sites; A1.1, A1.2, A2.1, A2.2, splice acceptor sites. (**B**) Linearized genomes were nucleofected in UT7/EpoS1 cells, which were fixed and stained to detect NS1 and VP1/2 at 48 hpt and 6 dpt to quantify gene expression by CLSM analysis. Supernatants were collected at 6 dpt and used to infect EPCs, from which DNA was extracted at 2 hpi, 48 hpi, and 6 dpi to allow measurement of viral DNA replication. (**C**) Representative images of UT7/EpoS1 cells nucleofected with the indicated pCK10 derivatives and stained to allow visualization of nuclei (blue) and NS1 or VP1/2 (green), acquired using a Nikon AX-R microscope equipped with a 20× objective as described in the Section 2. The percentage of NS1 (**D**) and VP1/2 (**E**) positive cells is shown as mean and standard deviation of the mean (SD) from at least four independent fields containing > 150 cells, along with the results of the two-way ANOVA test. *** = *p* ≤ 0.001; **** = *p* ≤ 0.0001. (**F**) Quantification of viral replication in EPCs infected with supernatants obtained at 6 dpt from UT7/EpoS1 cells nucleofected with the indicated linearized pCK10 plasmids. Data are shown as means ± SD from a single experiment performed in triplicate, along with the results of a two-way ANOVA test between the different time points. ** = *p* ≤ 0.01; *** = *p* ≤ 0.001; **** = *p* < 0.0001.

We have recently shown that the K177T substitution impairs NS1:IMPα interaction, resulting in partial mis-localization of the protein to the cytoplasm and inhibiting viral transcription in a minigenome system [22]. This suggested that NS1 nuclear localization is essential for viral genome replication. We therefore decided to verify this hypothesis in the context of a cloned, infectious viral genome. To this end, we mutated plasmid pCK10 [25] to introduce the K177T substitution within the NS1 coding sequence (Figure 1A). As a control, we also introduced a mutation to disrupt the first ATG within the NS1 coding sequence (NS1-ATG-KO). Subsequently, UT7/EpoS1 cells were nucleofected with linearized plasmid inserts, corresponding to B19V genomes obtained from wild-type and mutated pCK10 plasmids (Figure 1B), and cell cultures were maintained for a further 6 dpt. At 48 hpt and 6 dpt, aliquots of cells and supernatants were collected, and IIF assays were performed to monitor viral gene expression (Figure 1C–E). At 48 h and 6 dpt, both NS1 (Figure 1C,D) and VP1/2 (Figure 1C,E) could be detected in cells transfected with wild-type pCK10, although with different efficiency. Importantly, disruption of the first ATG within the NS1 coding sequence strongly reduced—but not abolished—the percentage of cells expressing NS1 (Figure 1D) while completely prevented expression of structural proteins VP1/2 (Figure 1E), suggesting that in the absence of the wild-type ATG, one or more shorter NS1 polypeptides could be translated using alternative downstream translation initiation sites, which can be recognized by the NS1 monoclonal antibody #892/5 (Supplementary Figure S1). On the other hand, the NS1:K177T substitution did not affect the number of cells expressing NS1 (Figure 1D) while strongly reducing the percentage of cells expressing VP1/2 (Figure 1E). Thus, impairing NS1 nuclear localization negatively affected the expression of B19V structural proteins. To test the effect of point mutations on the functional competence of the virus released in supernatants of cell cultures, we tested the ability of supernatants collected at 6dpt to infect EPCs. Following the addition of supernatants, containing 2.45–2.82 Log B19V geq/cell, cell cultures were maintained for a further 6 dpi, and aliquots of cells were collected 2 hpi, 48 hpi, and 6 dpi for qPCR analysis of viral replication (Figure 1F). Infection of EPCs with supernatants collected from UT7/EpoS1 nucleofected with B19V wild-type genome resulted in a one and two log increase in viral genome copy number at 48 hpi and 6 dpi as compared to 2 hpi, respectively (Figure 1F). In contrast, a significant decrease in viral genome copy number was measured for supernatants obtained from UT7/EpoS1 cells nucleofected with pCK10-NS1;K177T (Figure 1F), thereby demonstrating that NS1 nuclear import is important for viral replication. A similar reduction was observed using NS1-ATG-KO supernatants, indicating that full-length NS1 is essential for viral replication.

### 3.2. IVM Interferes with the IMPα:NS1-NLS Interaction

**Figure 2:**
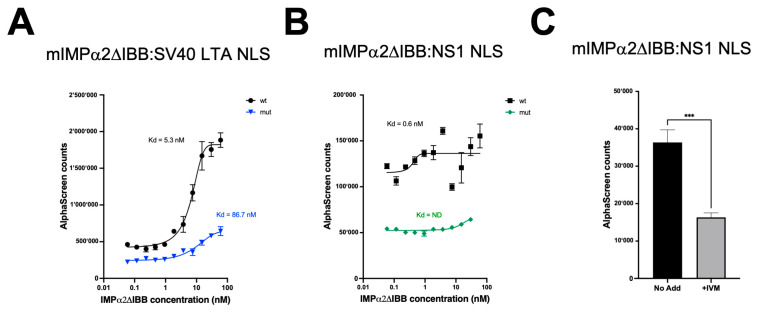
IVM inhibits the IMPα2ΔIBB:NS1 NLS interaction. Biotinylated peptides (30 nM) corresponding to (**A**) SV40 LTA NLS (SQHSTPPKKKRKV-132) and its inactive mutant derivative (SQHSTPPKtKRKV-132) or (**B**) B19V NS1 NLS (GACHAKKPRIT-182), and its inactive mutated derivative (GACHAtKPRIT-182) were incubated with increasing concentrations of his6-tagged recombinantly expressed mIMPα2ΔIBB (range 0–60 nM). The assay was quantitated on a PerkinElmer FusionAlpha plate reader, triplicate values were averaged, and titration curves were generated with GraphPad Prism. (**C**) AlphaScreen assay conducted as per 2A/B whereby biotinylated B19V NS1 NLS wt peptide (30 nM) was incubated with his6-tagged recombinantly expressed mIMPα2ΔIBB (20 nM) in the absence (left) or presence (right) of IVM (2.5 μM). Data are shown as mean + SD relative to three independent experiments. *p* value was determined by Student’s *t*-test. *** = *p* ≤ 0.001.

We have previously shown that treatment with the FDA-approved antiparasitic drug IVM at 10 μM could inhibit B19V viral replication in UT7/EpoS1 cells [22]. A possible explanation for IVM antiviral activity is its ability to interfere with nuclear transport mediated by IMPα [33]. We therefore adapted a previously established AlphaScreen protocol to monitor the effect of IVM on the IMPα:NS1-NLS interaction. To this end, we tested the ability of biotinylated peptides containing either B19V NS1 NLS sequence (NS1 NLS wt: GACHAKKPRIT-182) or the K177T derivative mutant impaired in NLS activity (NS1 NLS mut: GACHAtKPRIT-182) to bind to mouse (m)IMPα2ΔIBB (Figure 2). mIMPα2ΔIBB was utilized as this does not display auto-inhibition and can bind to NLS sequences strongly in the absence of IMPβ1. As a control, we also tested the binding of biotinylated peptides containing the NLS sequence from SV40 LTA (SV40 LTA NLS wt: SQHSTPPKKKRKV-132) and a mutant derivative thereof (SV40 LTA NLS mut: SQHSTPPKtKRKV-132). As expected, strong binding of the SV40 LTA NLS wt peptide to mIMPα2ΔIBB was observed (Kd ~5nM, see Figure 2A), which was dramatically reduced for the SV40 LTA NLS mut peptide (Kd ~87 nM). Importantly, strong and specific interaction was also detected for the NS1 NLS wt peptide binding to mIMPα2ΔIBB, while no specific binding was detected for the NLS1 NLS mut peptide (Figure 2B). Next, we tested the effect of IVM on the mIMPα2ΔIBB:NS1 NLS peptide interaction. To this end, the biotinylated B19V NS1 NLS wt peptide (60 mM) was mixed with mIMPα2ΔIBB (30 nM) in the absence or in the presence of IVM (10 μM). Importantly, the addition of IVM caused a significant reduction in AlphaScreen counts (Figure 2C). Therefore, IVM decreased the mIMPα2ΔIBB:NS1 NLS interaction.

### 3.3. IVM Inhibits B19V Replication in a Dose-Dependent Manner

**Figure 3:**
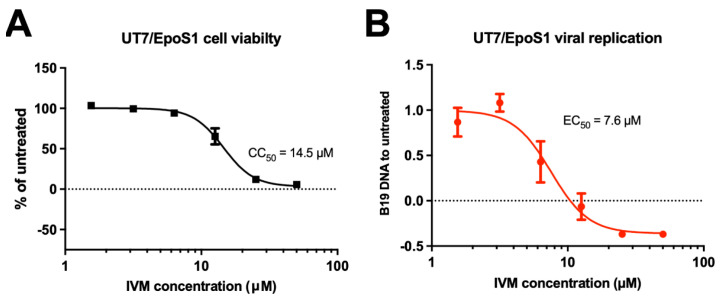
IVM inhibits B19V replication in a dose-dependent fashion. (**A**) UT7/Epo-S1 were treated for 6 h with increasing concentrations of IVM and then incubated for a further 48 h; cell viability was measured by using the alamarBlue™ HS Cell Viability Reagent. (**B**) UT7/Epo-S1 were treated for 6 h with increasing concentrations of IVM then infected with B19V and further incubated with IVM for 48 hpi; viral DNA replication was assessed by qPCR as the variation in the amount of viral DNA at 48 hpi compared to 2 hpi. Data were normalized to untreated cells and shown as mean ± SD relative to three independent experiments. Half maximal cytotoxicity concentration (CC_50_) and half maximal effective concentration (EC_50_) were calculated as described in the Section 2.

We therefore decided to perform a dose response curve for the antiviral activity of IVM against B19 replication. To this end, UT7/Epo-S1 cells, either untreated or treated for 6 h in the presence of different concentrations of IVM (range 0–50 μM), were infected with B19V for 2 h in the absence of IVM. After 2 washes in PBS to remove residual viral particles, cells were further incubated in the presence or absence of different concentrations of IVM for 48 h. Viral DNA replication was evaluated by quantifying DNA levels extracted from cells at 2 h and 48 h post-infection. In parallel, uninfected cells were either incubated with increasing concentrations of IVM or left untreated, and cell viability was measured (Figure 3). Our data indicated that IVM did cause significative toxicity at concentrations above 12.5 μM, with a CC_50_ of 14.5 μM (Figure 3A). Importantly, IVM treatment showed strong inhibition of B19V DNA replication, starting from 6 μM, with an EC_50_ of 7.6 μM (Figure 3B). Therefore, despite the limited SI (Table A1), IVM-mediated pharmacological inhibition of IMPα-dependent NS1 nuclear import can hinder B19V replication in a dose-dependent fashion.

### 3.4. AVMs Inhibit the IMPα:NS1-NLS Interaction with Comparable Potency as IVM

**Figure 4:**
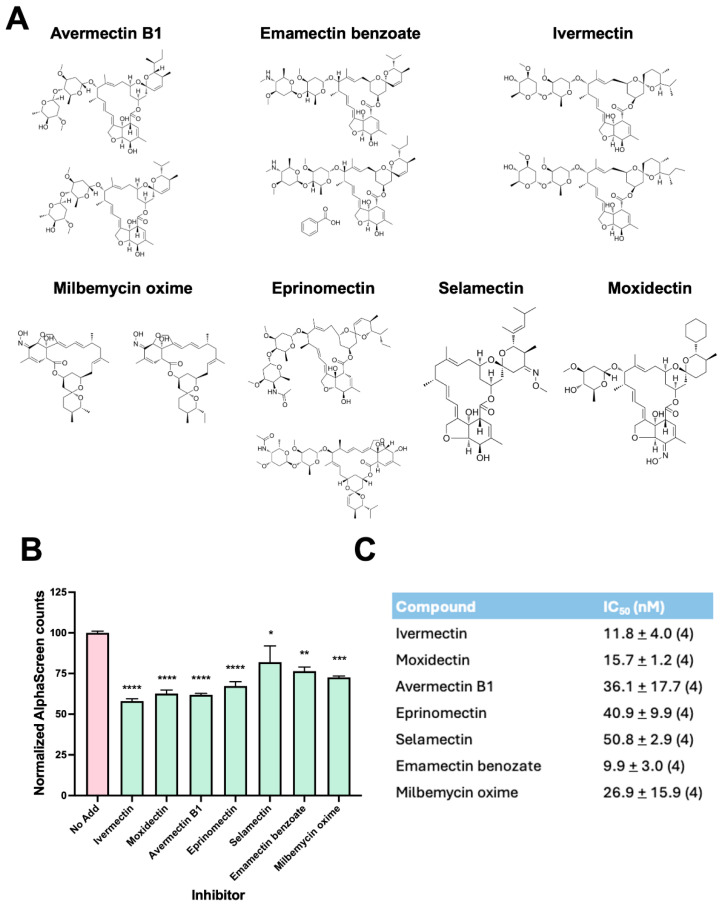
AVMs impair the mIMPα2ΔIBB:NS1 NLS interaction. (**A**). Chemical structure of the AVMs used in this study. (**B**) AlphaScreen assay conducted as per Figure 2C whereby biotinylated B19V NLS peptide (30 nM) was incubated with his6-tagged recombinantly expressed mIMPα2ΔIBB (20 nM) in the absence (No Add) or presence of the indicated compounds (5 μM). Results are for % AlphaScreen counts compared to No Add, shown as means + standard error of the mean (SE) relative to four independent experiments. *p* value was determined by 1-way ANOVA. **** = *p* < 0.0001; *** = *p* < 0.001; ** = *p* < 0.01; * = *p* < 0.05. (**C**) Average IC_50_ values determined from titration of each compound (0–5 μM) for 4 independent experiments are shown, along with the SD.

In UT7/EpoS1 cells, the calculated SI for IVM was 1.9, which is far from ideal for therapeutic applications. IVM belongs to the class of AVMs, a group of macrocyclic lacton derivatives with similar structural features endowed with anthelmintic and insecticidal properties and therefore widely used as pesticides and anti-anthelmintic drug [34]. Since certain AVMs have been proven superior to IVMs in inhibiting severe acute respiratory syndrome coronavirus 2 (SARS-CoV-2) viral replication [35,36], we therefore reasoned that they might inhibit the IMPα:B19V NS1-NLS interaction similarly to IVM, but prove more selective in cell culture as antivirals. Therefore, we screened a panel of AMVs for their ability to inhibit the IMPα:NS1-NLS interaction in vitro, using our recently described AlphaScreen assay, including IVM as a control (Figure 4A). To this end, recombinant IMPα2ΔIBB was incubated with a biotinylated NS1-NLS peptide in the presence of each compound at a 5 μM final concentration. Importantly, all tested AVMs inhibited the IMPα:NS1-NLS interaction to a similar extent as compared to IVM (Figure 4B). Dose-dependent inhibition assays carried out using increasing concentrations of compounds revealed that all tested AMSs could inhibit the IMPα:NS1-NLS in the nanomolar range (Figure 4C).

### 3.5. Eprinomectin Inhibits B19V Replication More Potently than IVM in UT7/EpoS1 Cells

**Figure 5:**
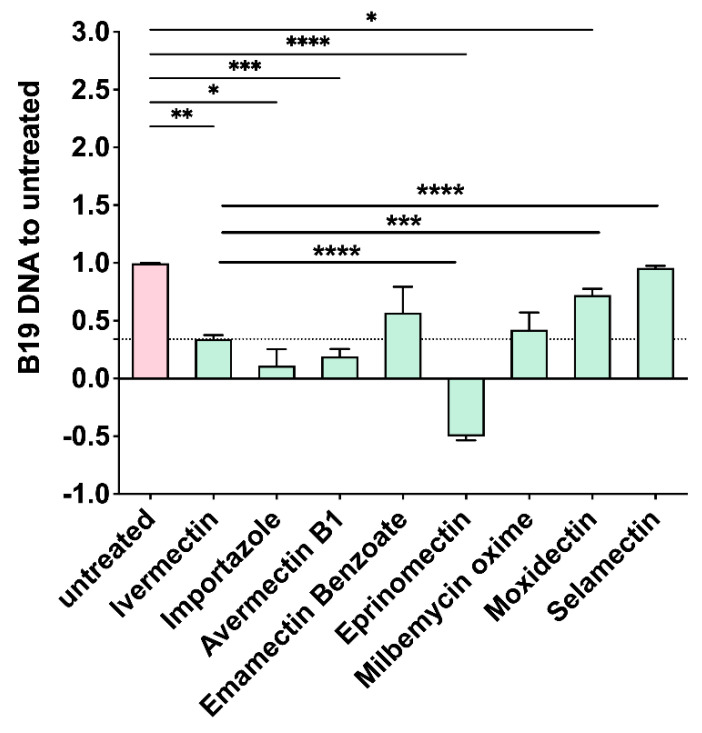
AVMs impair B19V replication. UT7/EpoS1 cells were either left untreated or treated for 6 h with the indicated compounds (10 µM) then infected with B19V as described and incubated with media containing the indicated compounds for a further 48 h. Viral DNA replication was assessed by qPCR as the variation in the amount of viral DNA at 48 hpi compared to 2 hpi. Data relative to three independent experiments were normalized to untreated cells and shown as mean + SD. Histogram bars express these data as variations, normalized to the replication in the absence of compounds (set as 1.0). The dashed horizontal line corresponds to viral replication in the presence of IVM. Results of statistical analysis relative to the comparison of the effect of each treatment as compared to untreated cells or IVM using the Welch and Brown-Forsythe one-way ANOVA test. *: *p* ≤ 0.05; **: *p* ≤ 0.01; ***: *p* ≤ 0.001; ****: *p* ≤ 0.0001.

Since AVMs interfered with the NS1 NLS:IMPα interaction, we decided to investigate their effect on B19V replication. To this end, UT7/EpoS1 cells were either untreated or treated for 6 h in the presence of 10 μM AVMs, subsequently infected with B19V for 2 h in the absence of compounds, and further incubated for 48 h to allow for viral replication. IVM and Importazole, a small molecule interfering with IMPβ1-dependent nuclear import [37], were also used as positive controls. As expected, both IVM and Importazole inhibited viral replication. Importantly, all compounds except Moxidectin and Selamectin inhibited viral replication to similar or higher levels as compared to IVM (Figure 5).

**Figure 6:**
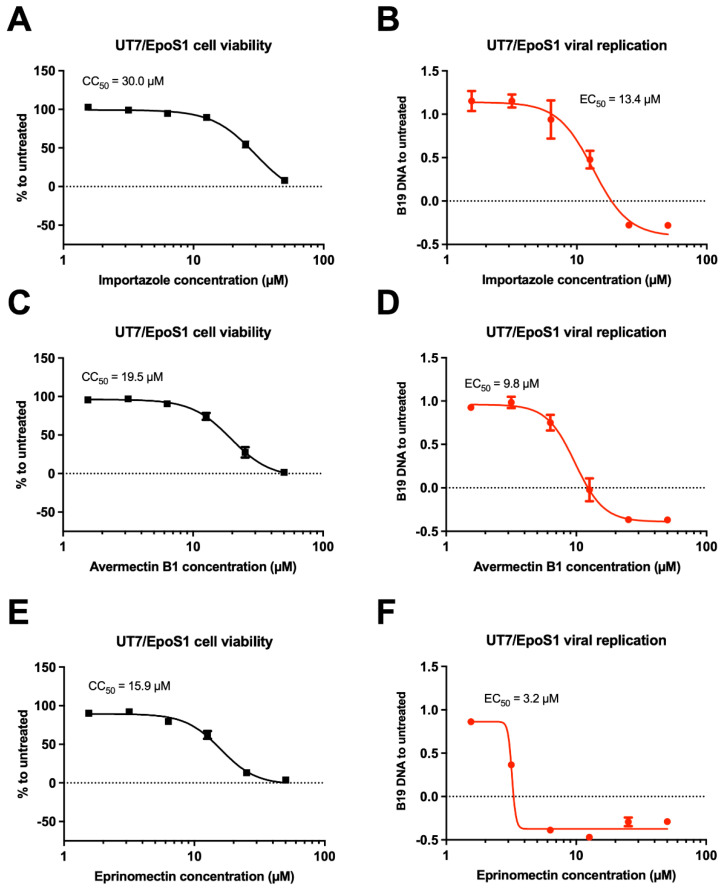
Eprinomectin inhibits B19V replication more selectively than IVM. UT7/EpoS1 were either treated with increasing concentrations of indicated compounds for 48 h and then cell viability measured by using the alamarBlue™ HS Cell Viability Reagent (**A**,**C**,**E**) or treated for 6 h with increasing concentrations of the indicated compounds then infected for 2 h with and incubated for further 48 hpi to allow quantification of viral DNA replication as described (**B**,**D**,**F**). Data were normalized to untreated cells and shown as mean ± SD relative to three independent experiments. Half maximal cytotoxicity concentration (CC_50_) and half maximal effective concentration (EC_50_) were calculated as described in the Section 2.

Importazole, Avermectin B1, and Eprinomectin inhibited viral replication more than IVM. We therefore decided to test their antiviral properties and cellular toxicity by dose–response assays, as described above for IVM. Importazole was well tolerated by UT7/EpoS1 cells, with a CC_50_ of 30.0 μM (Figure 6A). Avermectin B1 and Eprinomectin were slightly more cytotoxic, with CC_50s_ of 19.5 (Figure 6C) and 15.9 μM (Figure 6E), respectively. On the other hand, Eprinomectin was more potent as compared to the other inhibitors tested against B19V replication (Figure 6F), with an EC_50_ of 3.2 μM, considerably lower than those measured for Importazole (13.4 μM; Figure 6B) and Avermectin B1 (9.8 μM; Figure 6D) and resulting in a SI of 5.0 (Figure 6, Table A1).

### 3.6. High Cytotoxicity of Eprinomectin in EPCs

**Figure 7:**
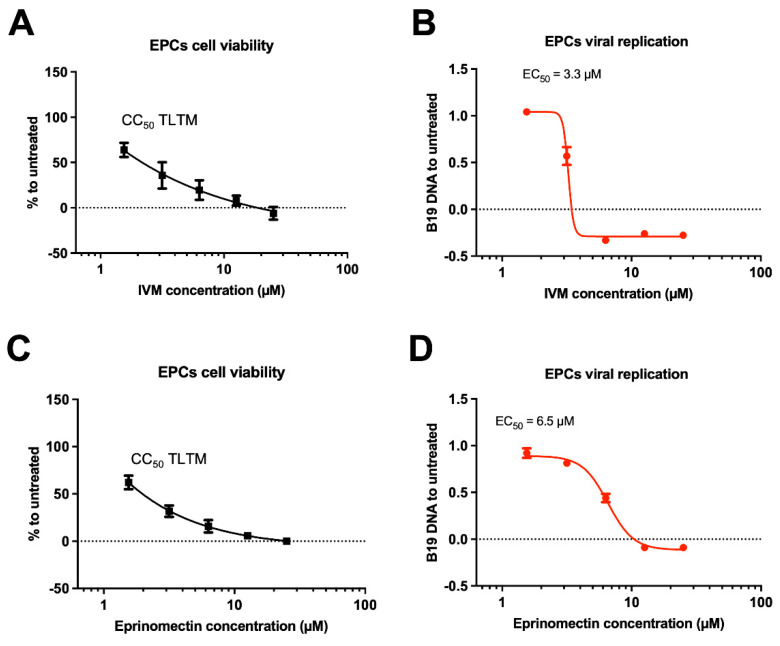
IVM and Eprinomectin exhibit high cytotoxicity in EPCs. EPCs were either treated with increasing concentrations of indicated compounds for 48 h to allow measurement of cell viability using a WST-8 based assay (**A**,**C**) or treated for 6 h with increasing concentrations of the indicated compounds then infected for 2 h and further incubated for 48 hpi to allow quantification of viral DNA replication as described (**B**,**D**). Data were normalized to untreated cells and shown as mean ± SD relative to three independent experiments. Half maximal cytotoxicity concentration (CC_50_) and half maximal effective concentration (EC_50_) were calculated as described in the Section 2. TLTM: too low to measure.

B19V primarily replicates in vivo in EPCs in the bone marrow or the livers of adults and fetuses, respectively [9]. EPCs are highly proliferating cells and extremely sensitive to a number of otherwise highly selective antivirals [38]; thus, we sought to compare the selectivity of IVM and Eprinomectin as B19V inhibitors in EPCs in vitro by testing their antiviral properties and cell toxicity as above (Figure 7). Our results indicate that, despite both compounds could inhibit B19V replication with similar potency in EPCs, they were extremely toxic at every concentration tested (Figure 7). Therefore, neither compound is endowed with the desired selectivity. Our results suggest that inhibition of the NS1:IMPα interaction impairs B19V replication. However, the poor selectivity of available nuclear transport inhibitors causes significant toxicity in highly proliferating EPCs.

## 4. Discussion

Antivirals are an unmet need to contrast B19V infections, which can be life-threatening under several circumstances, including infection of immunocompromised or anemic patients and vertical transmission. Promising antivirals identified so far include the acyclic nucleoside phosphonate cidofovir [39] and its lipid conjugate brincidofovir [40], both exhibiting broad-spectrum antiviral activities against all DNA viruses. The development of highly specific antivirals has been hampered by the paucity of structural and functional information regarding key viral proteins [21,39,41,42]. NS1 is emerging as a potential target of therapeutic intervention, playing a crucial role during viral genome replication within the host cell nucleus. Indeed, NS1’s ability to recognize the origin or B19V replication and cleave it at the trs sequence is essential for the start of the strand amplification via the rolling hairpin mechanism [15,43], leading to viral replication, with molecules targeting its endonuclease activity being endowed with antiviral properties [44]. In this context, our recent characterization of B19V NS1 NLS interaction with IMPα suggested an interesting target of potential therapeutic intervention, further strengthened by the evidence that the FDA-approved antiparasitic drug IVM inhibited viral replication at 10 μM [22].

Our novel data reported here confirm the crucial role of NS1 nuclear import in viral replication. First of all, the NS1 K177T substitution, which causes a strong defect in IMPα binding and nuclear targeting [22], reduced expression of structural proteins VP1/2 (Figure 1D), and completely abolished B19V replication in cell culture when introduced in the context of a full replication competent viral genome (Figure 1E). Secondly, drugs interfering with IMPα/β dependent nuclear import, such as IVM—which targets the interaction between IMPα and IMPβ [24] and Importazole—which targets the interaction of IMPβ with RanGTP [37], also inhibit viral replication in a dose-dependent manner (Figure 3 and Figure 6). Thirdly, other compounds structurally related to IVM and similarly able to impair the NS1:IMPα interaction (see Figure 4) also impaired viral replication in cell culture, with Importazole, Avermectin B1, IVM, and Eprinomectin being the most effective (Figure 5 and Figure 6). In UT7/EposS1 cells, the SI for IVM (1.9) was in line with those reported for several other viruses, including SARS-CoV-2, Dengue virus (DENV), Human Adenovirus and West Nile virus, but markedly lower than that described for Yellow Fever virus [35,45,46,47,48,49]. Indeed, besides its ability to impair IMPα/β nuclear import, IVM has also been shown to potently inhibit flavivirus helicase activity, being therefore endowed with a dual potential mechanism of action [46]. IVM has been shown to induce cell cycle arrest, apoptosis, and autophagy, presumably through mitochondrial pathways, further limiting its potential use in vivo [50]. Accordingly, despite IVM and Importazole have been successfully used to inhibit the replication of several viruses in cell culture [22,35,45,47,48,49,51,52,53,54,55,56], their activity in animal models and humans has not proved satisfactory. This has been hypothesized to depend on pharmacokinetic issues, with IVM concentrations achievable in the site of viral replication after administration of non-toxic dosages being too low to allow sufficient antiviral activity against Zika virus in a murine system [57]. Similarly, despite IVM proving highly effective in inhibiting DENV replication in cell culture [46,47], and in Aedes Albopictus [58], elevated toxicity in AG129 mice hampered its antiviral activity. Interestingly, such an issue was circumvented by lowering IVM dosage and co-administration with atorvastatin, an agent similarly capable of reducing nuclear import of NS3 [59]. In the case of B19V, our data show that their toxicity is further increased in EPCs (Figure 7), as expected by such a highly proliferating cellular population. Accordingly, several compounds, including NS1 endonuclease inhibitors [44], as well as a number of currently approved FDA antivirals exhibited high toxicity when tested in EPCs [38,60], highlighting the specific relevance of such specialized cellular systems in the investigation of the viral replicative cycle and virus-cell interactions.

Additionally, it is worth mentioning that we could detect a few positive cells by IIF upon nucleofection of UT7/EpoS1 cells with pCK10-NS1-ATG-KO (Figure 1D). This suggests the possibility that B19V, similarly to Adeno-associated virus 5, exploits alternative translation initiation sites to produce additional forms of its main replicative protein, possibly endowed by distinct functions during virus replication [61,62]. Future work in our laboratory will investigate this intriguing possibility.

## 5. Conclusions

Our study clearly identifies NS1 nuclear transport and its interaction with IMPα as potential targets of therapeutic intervention. However, given the high sensitivity of EPCs to current inhibitors of nuclear import, more specific inhibitors should be identified.

## Data Availability

The original data presented in the study are openly available in Research data UNIPD at https://researchdata.cab.unipd.it/1483/ (accessed on 29 January 2025).

## Supplementary Materials

The following supporting information can be downloaded at https://www.mdpi.com/article/10.3390/v17020220/s1, Figure S1: Potential alternative translation initiation sites for B19V NS1.

## Appendix A

**Table A1:** Antiviral properties of indicated compounds. The half-maximal cytotoxic concentration (CC_50_), half-maximal effective concentration (EC_50_), and selectivity index (SI) of indicated compounds in UT7/EpoS1 cells or EPCs are shown. Data are mean along the 95% confidence intervals. TLTM: too low to measure; N/A: not assessed.

Compound	UT7/EpoS1			EPCs		
	CC_50_ (μM; 95% CI)	EC_50_ (μM; 95% CI)	SI	CC_50_ (μM; 95% CI)	EC_50_ (μM; 95% CI)	SI
IVM	14.5 (12.1 to 17.4)	7.6 (5.1 to 11.1)	1.9	TLTM	3.3 (very wide)	N/A
Eprinomectin	15.9 (13.2 to 19.1)	3.2 (very wide)	5.0	TLTM	6.5 (6.0 to 7.1)	N/A
Importazole	30.0 (22.9 to 39.3)	13.4 (9.8 to 18.2)	2.3	N/A	N/A	N/A
