# Supplementary figures and images for: Avermectins Inhibit Replication of Parvovirus B19 by Disrupting the Interaction Between Importin α and Non-Structural Protein 1
Source: Viruses. 2025 Feb 3;17(2):220. doi: 10.3390/v17020220 (PMC11860776; doi:10.3390/v17020220)

## Slide 1
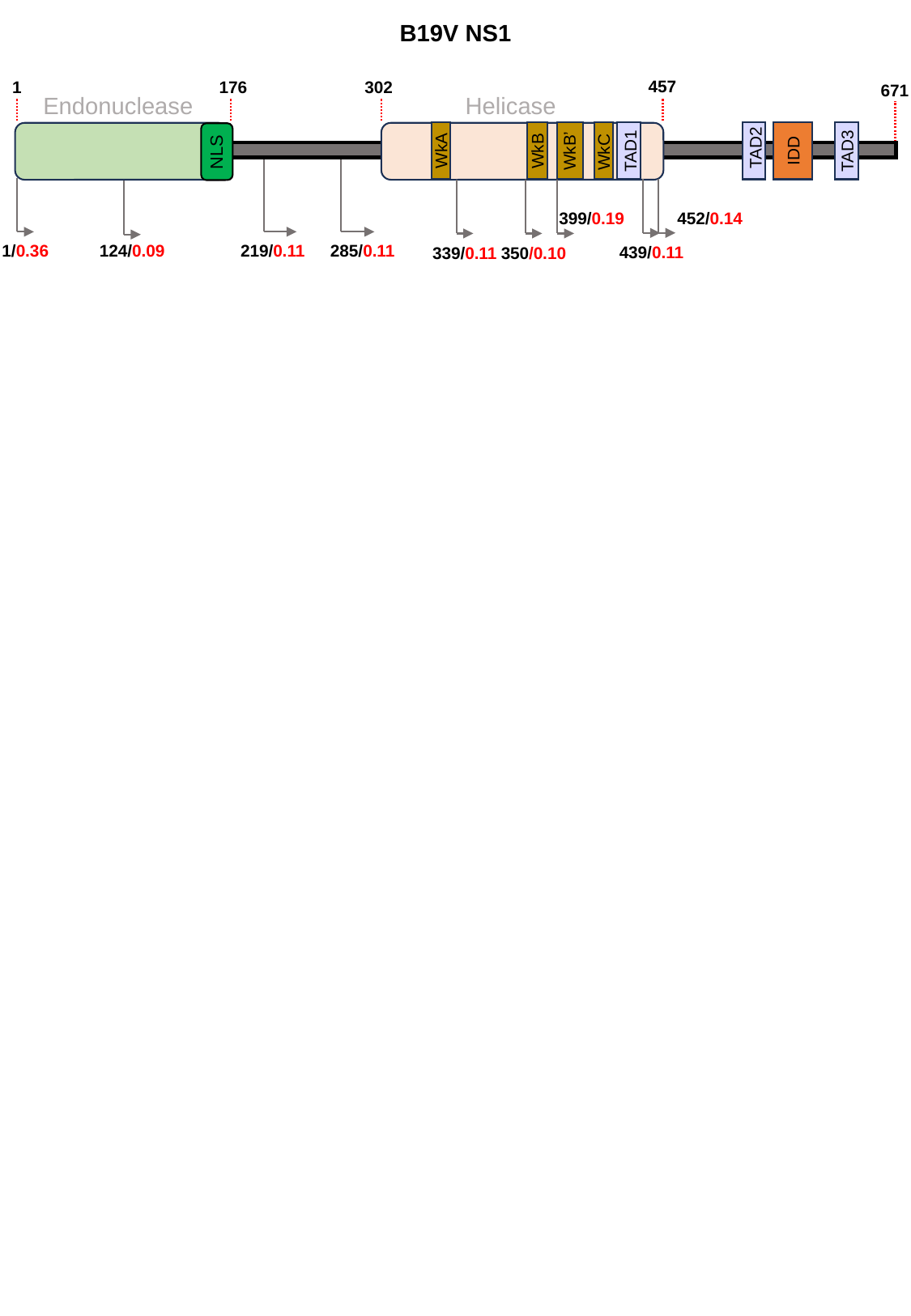

B19V NS1
457
1
176
302
671
Endonuclease
Helicase
TAD2
WkA
WkB
WkB’
WkC
TAD1
IDD
TAD3
NLS
399/0.19
452/0.14
1/0.36
124/0.09
219/0.11
285/0.11
439/0.11
339/0.11
350/0.10

Supplement: Supplementary file 1 [file viruses-17-00220-s001.zip › viruses-3434695-supplementary.pptx]
